# Electrochemical Behaviour of Tinidazole at 1,4-Benzoquinone Modified Carbon Paste Electrode and Its Direct Determination in Pharmaceutical Tablets and Urine by Differential Pulse Voltammetry

**DOI:** 10.1155/2017/8518707

**Published:** 2017-11-07

**Authors:** Yosef Nikodimos, Beyene Hagos

**Affiliations:** Department of Chemistry, Woldia University, P.O. Box 400, Woldia, Ethiopia

## Abstract

A simple and highly sensitive electrochemical method based on a 1,4-benzoquinone modified carbon paste electrode (1,4-BQMCPE) was described for the determination of tinidazole (TDZ). In Britton Robinson buffer solution, TDZ yields well-defined irreversible reduction peak at −0.344 V on a 1,4-BQMCPE. Compared with that on a bare CPE, the reduction peak of TDZ increased significantly on the modified CPE and the effects of different parameters on the voltammetric responses were also investigated. Differential pulse voltammetric method was proposed and optimized for TDZ determination and its reductive peak current response at 1,4-BQMCPE was found to show linear dependence on the concentration of TDZ in the range of 1.0 × 10^−6^ to 5.0 × 10^−4^ M with a linear regression equation, correlation coefficient, limit of detection (LOD), and limit of quantification (LOQ) of *I*_PC_ (*μ*A) = 0.19958 + 0.02657*C* (*μ*M), 0.99486, 1.10 × 10^−7^ M, and 3.77 × 10^−7^, respectively. Excellent recovery results for spiked TDZ in pharmaceutical tablet samples ranging within 97.44–97.51% and in urine ranging within 95.37–96.91% were observed. The selectivity of the method for TDZ was further studied in the presence of selected potential interferents and confirmed the potential applicability of the developed method for the determination of TDZ.

## 1. Introduction

TDZ, C_8_H_13_N_3_O_4_S, which is 1-(2-ethyl sulfonyl ethyl)-2-methyl-5-nitro-imidazole derivative, an antiparasitic drug, is used as an antiprotozoal drug (molecular weight: 247.273 g/mol). It is highly effective for bacterial [1–3] and protozoan [3] infections and is available in the tablet form.

TDZ is an antiparasitic drug used against protozoan infections, bacterial infections, infections of the blood, lungs infection, chest infections, skin infections, womb lining infections, gums infection, vaginal or stomach infections, and others. It is widely known throughout the world as a treatment for a variety of amoebic and parasitic infections. A large body of clinical data exists to support its use as a treatment for amebas, giardia, and vaginal trichomoniasis [1–5].

Taking into account its importance, the determination of TDZ has a great importance. Many methods have been developed and applied in the detection of TDZ like electrochemical techniques [6–10], titrimetric [11], spectrophotometry [11–17], and HPLC [12, 16, 18–22].

Since most of the conventional methods reported need trained personnel to operate and are expensive and not environmentally friendly, the development of another alternative electrochemical method which is selective, sensitive, cheap, and environmentally friendly is necessary.

In recent years, polymer-modified electrodes have received attention due to their good stability, reproducibility, increase in active sites, homogeneity in electrochemical deposition, and strong adherence to electrode surface [23]. The benzoquinone modified electrodes had shown remarkable advantages from their low noise levels and higher sensitivity [24].

This paper describes voltammetric method for determination of TDZ via its reduction at a 1,4-benzoquinone modified carbon paste electrode (1,4-BQMCPE). The electrode process was investigated by cyclic voltammetry (CV) and differential pulse voltammetry (DPV). The influence of different experimental parameters such as pH, accumulation time, and scan rate was investigated to optimize the proposed method. The method is successfully applied for the determination of TDZ in pharmaceutical tablets and urine, and the results obtained were compared with earlier reported method.

## 2. Experimental Part

### 2.1. Apparatus and Reagents

BAS 100B electrochemical analyzer [Bioanalytical Systems (BAS), USA] connected to a personal computer was used for the voltammetric measurements. A three electrodes' system consisting of 1,4-BQMCPE as working electrode, platinum coil as auxiliary electrode, and Ag/AgCl as reference electrode was used. The pH of the buffer solutions was measured with Jenway model 3310 pH meter. An electronic balance (Denver instrument) was used for measuring mass of different chemicals and samples. A magnetic stirrer with a hot plate was used for stirring during pH adjustments.

Standard TDZ (Emmelen Biotech Pharmaceuticals Limited) and TDZ tablet of different brands (APF and EPHARM) were used. Graphite powder (BDH-Laboratory Supplies, Poole, England), paraffin oil (Abron Chemicals), boric acid (BIO-lab laboratories LTD), phosphoric acid (Veeni Chemicals), glacial acetic acid and NaOH (supplied by Blulux laboratories reagent), HCl (BDH Limited, Poole, England), and 1,4-benzoquinone (Riedel-De Haen, Germany) were used in the experiment. Distilled water was used throughout the work.

### 2.2. Procedures

#### 2.2.1. Preparation of TDZ Standard Solutions

**Scheme 1:**
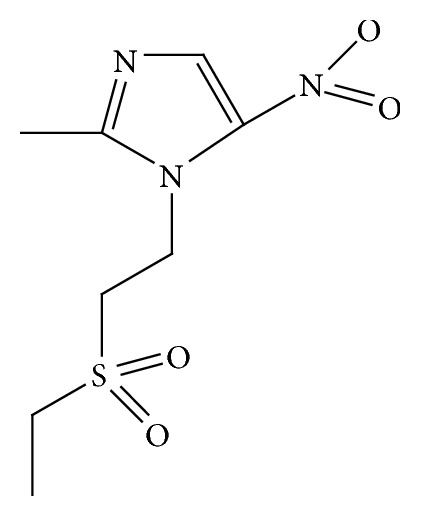
Structure of tinidazole.

0.01 molar stock solution of TDZ was prepared by dissolving 0.247 grams of standard TDZ in 100 mL of 5% of ethanol with water. Tinidazole (see Scheme 1) working solutions were prepared by diluting the stock solution with the BRB buffer solutions of the required pH. The supporting electrolyte Briton Robinson buffers (BRB) in the pH range 2.0–7.0 were prepared from H_3_BO_4_, CH_3_COOH, and H_3_PO_4_ each with 0.04 molar in distilled water. 1.0 mol L^−1^ NaOH and 1.0 mol L^−1^ HCl solutions were used to adjust the pH of the buffer solution.

#### 2.2.2. Preparation of Pharmaceutical Tablet Samples

TDZ tablets (EPHARM and APF) were purchased from pharmacy. Tablets (labeled as 500 mg TDZ/tablet) of pharmaceutical formulations were accurately weighed and finely powdered in a porcelain mortar. 0.296 grams of this powder, corresponding to a stock solution of concentration 0.01 molar, was weighed and transferred into a 100 mL flask and dissolved with 5% of ethanol solution. The tablet solutions were filtrated using a Whatman® filter paper. Then, 50.0 and 90 *μ*M sample solutions were prepared from the stock solution using 0.04 molar BRB solutions for each TDZ tablet brand.

#### 2.2.3. Preparation of TDZ Solution in Urine

The urine sample was taken from a volunteer healthy individual immediately before the experiments. The urine sample was suction filtrated using a 0.45 *μ*m pore size filter paper. The filtrate was then diluted with pH 5 BRB solution in a 1 : 4 volume ratio. Then, 90 *μ*M solutions were prepared from the different brands of TDZ tablets using the diluted filtrated urine.

#### 2.2.4. Preparation of Working Electrodes

Unmodified carbon paste electrode (CPE) (100 mg) was prepared by mixing graphite powder with paraffin oil. The composition of the paste was 75% (w/w) graphite powder and 25% (w/w) paraffin oil. The mixture was homogenized with mortar and pestle for 30 minutes and allowed to rest for 24 hours. The homogenized paste was packed into the tip of a plastic syringe (3 mm diameter, 7 mm deep). A copper wire was inserted from the backside of the syringe to provide electrical contact. Then the surface of the electrode was smoothed against a smooth white paper with a light manual pressure until a shiny surface is emerged.

Modified carbon paste (100 mg) was prepared by mixing graphite powder with 1,4- benzoquinone in paraffin oil. To 10 mg of 1,4-benzoquinone and 70 mg of carbon powder initially mixed with a mortar and pestle for 5 minutes, 23 *μ*L (20 mg) of paraffin oil was added and thoroughly mortared together for 30 minutes. The resulting paste was packed into the tip of the syringe by extruding a small amount of paste from the tip of the previously prepared unmodified carbon paste electrode.

#### 2.2.5. Electrochemical Procedure

Both the Ag/AgCl reference and Pt auxiliary electrodes were rinsed with distilled water prior to each measurement. The surface of the 1,4-BQMCPE was also smoothed manually against a smooth white paper. Voltammetric measurements were recorded using 1,4-BQMCPE working electrode after a stable voltammogram was obtained in BRB solution.

Cyclic voltammetric measurements were recorded and the influence of pH on the net peak current of TDZ was investigated over pH range of 2.0–7.0. The effect of scan rate on both the reductive peak current and peak potential was investigated in the range 20 to 200 mVs^−1^. Furthermore, differential pulse voltammetry was used for the quantitative determination of TDZ in pharmaceutical samples. After a calibration curve was constructed using external standard addition of TDZ solutions of different concentrations, the regression equation was used for the determination of the TDZ content in tablets of different brands. Recovery results of spiked standard TDZ in tablet solutions and urine and interference study results were used to validate the applicability of the developed method for the determination of TDZ in pharmaceutical formulations and urine. All experiments were carried out at room temperature.

## 3. Results and Discussion

### 3.1. The Cyclic Voltammetric Investigation of TDZ at 1,4-BQMCPE

#### 3.1.1. Electrochemical Behaviour of TDZ at 1,4-BQMCPE

**Figure 1:**
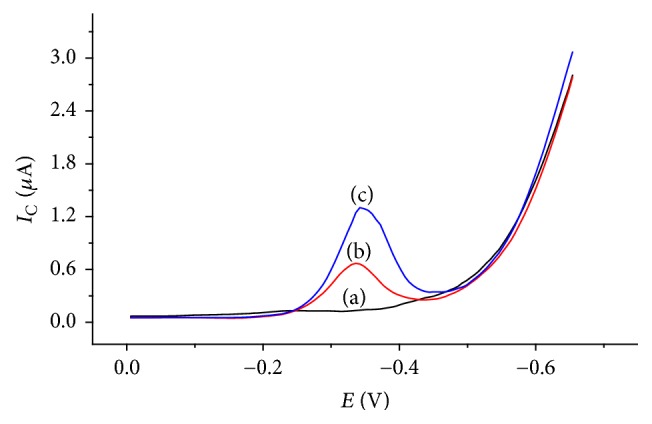
Cyclic voltammograms of (a) CPE in BRB solution containing no TDZ, (b) CPE in BRB solution containing 0.5 mM TDZ, and (c) 1,4-BQMCPE in BRB solution containing 0.5 mM TDZ; scan rate: 100 mV s^−1^; potential window: 0 to −700 mV.

The electrochemical behaviour of TDZ was studied using cyclic voltammetry in BRB solution.  Figure 1 shows the cyclic voltammograms of 0.5 mM TDZ using CPE (curve (b)) and 1,4-BQMCPE (curve (c)). The voltammograms of CPE and 1,4-BQMCPE in the buffer solution containing TDZ showed a distinct irreversible reductive peak which is absent at the voltammogram recorded in the absence of TDZ (curve (a)).

Comparing the two results, there was excellent improvement in the voltammograms when 1,4-BQMCPE was used. The response in the cyclic voltammograms during the experiment revealed that, in case of unmodified carbon paste electrode, the reduction peak current was observed at 0.680 *μ*A while, in the case of the 1,4-BQMCPE, the reduction peak was observed at 1.31 *μ*A.

#### 3.1.2. Effect of Scan Rate

**Figure 2:**
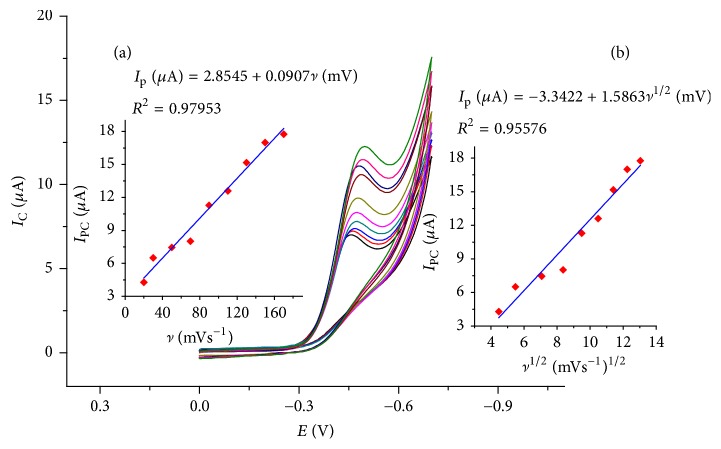
Cyclic voltammograms of 1,4-BQMCPE in BRB containing 0.5 mM TDZ at various scan rates (20, 40, 60, 80, 100, 125, 150, 175, and 200 mV/s, resp.). Inset: plot of variation of *I*_PC_ versus (a) scan rate and (b) square root of scan rate.

The effect of scan rate on the electrochemical behaviour of TDZ was also investigated at various scan rates (Figure 2). The redox peak current increased linearly with the square root of the scan rate and the scan rate in the range of 20–200 mV/s. The regression equations can be expressed as *I*_p_ (*μ*A) = −3.3422 + 1.5863*v*^1/2^ (mV) (*R*^2^ = 0.95576) and *I*_p_ (*μ*A) = 2.8545 + 0.0907*v* (mV) (*R*^2^ = 0.97953), which indicated that the electrochemical reaction of TDZ at 1,4-BQMCPE is adsorption controlled process in the selected scan rate range.

The number of electrons transferred on the electrode process (*n*) for adsorption controlled irreversible process can be estimated using the following equations [25, 26]:

(1) IPC=αnn2F2AΓν2.718RT,

(2) EP−EP/2=1.85RTαnF V=0.048αn Vat  25C°,

(3) Γ=QnFA,

where *I*_PC_ is cathodic peak current, *ν* is scan rate, Γ is surface concentration of the electroactive species (mol cm^−2^), *A* is the electrochemical active area (cm^2^), *R* is the universal gas constant (8.314 J K^−1^ mol^−1^), *T* (K) is the Kelvin temperature, *F* is the Faraday constant (96485 C mol^−1^), *E*_P_ is peak potential, *E*_P/2_ is half-peak potential, and *Q* is charge consumed obtained from integral of peak area. Using (2) (*αn*) was estimated from (2) to about 2.096 for a scan rate of 100 mV. Substituting the Γ term of (3) into (1) a new relation for *n* is obtained:

(4) $$\begin{array}{c} n=\frac{2.718I_{PC}RT}{\left(\alpha n\right)FQ\nu }. \end{array}$$

Using (4) the number of electrons transferred in the electrode reaction (*n*) was calculated to be 4.413 which indicated that by approximation four electrons were involved in the reduction of TDZ on the 1,4-BQMCPE, a comparable result with earlier reported works [10]. The value of *α* is thus calculated to be 0.475 for TDZ still confirming the irreversibility of the reduction of TDZ on 1,4-BQMCPE.

**Figure 3:**
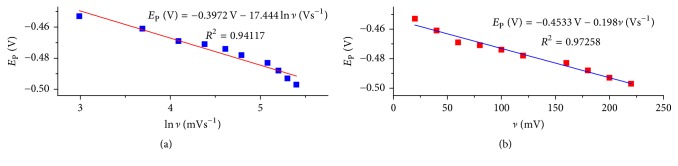
Plot of cathodic peak potential (*E*_P_) versus (a) ln of scan rate and (b) scan rate.

Linear dependence of peak potential (*E*_P_) against the logarithm of scan rates (ln⁡*ν*) was observed with a linear equation and correlation coefficient of *E*_P_ (V) = −0.3973 V − 17.0444ln⁡*ν* (Vs^−1^) and *R*^2^ = 0.94117, respectively (Figure 3(a)). For an irreversible cathodic reaction, the equation used to calculate standard rate constant is [27]

(5) $$\begin{array}{c} E_{P}=E^{o}+\frac{RT}{\alpha nF}\ln\left(\;\frac{RTK_{o}}{\alpha nF}\;\right)-\frac{RT}{\alpha nF}\ln\nu , \end{array}$$

where *E*_P_ is peak potential, *E*^*o*^ is the formal potential, *α* is the transfer coefficient, *K*_*o*_ (s^−1^) is the electrochemical rate constant and the other parameters have their usual meanings.

The value of *E*^*o*^ which was obtained from the intercept of the *E*_P_ versus *v* plot (Figure 3(b)) [28] was found to be −0.4533 V. The value of *K*_*o*_ evaluated from the intercept of the plot of *E*_P_ versus ln⁡*v* (Figure 6(a)) as the equation below was calculated to be 8,104.2126 s^−1^.

(6) $$\begin{array}{c} E^{0}+\frac{RT}{\alpha nF}\left[\;\ln\left(\;\frac{RTK_{o}}{\alpha nF}\;\right)\;\right]=-0.3972\;V. \end{array}$$

#### 3.1.3. Effect of pH

**Figure 4:**
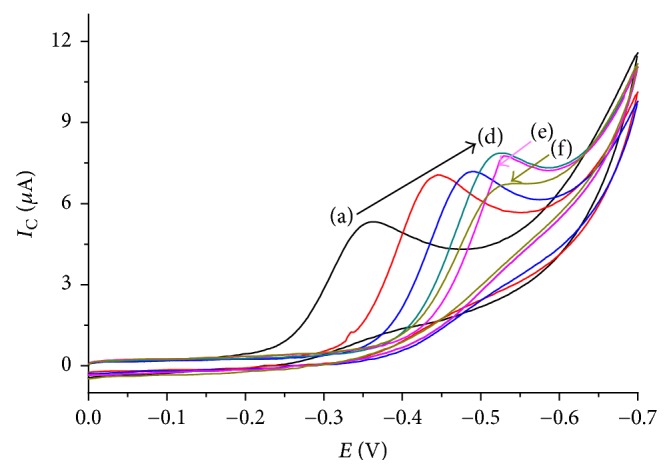
Cyclic voltammograms of 0.5 mM TDZ in BRB solution of different pH values ((a)–(f): 2.0, 3.0, 4.0, 5.0, 6.0, and 7.0, resp.). Scan rate: 100 mV s^−1^.

The effect of pH on the current response of 1,4-BQMCPE in 0.5 mM of TDZ was investigated in the pH range from 2.0 to 10.0 by cyclic voltammetry and the results were shown as in Figure 4.

Figure 4 represents only cyclic voltammograms of 0.5 mM TDZ in the region where its reduction is pH dependent (pHs 2.0–7.0). A well-defined irreversible cathodic peak was observed in the entire buffer system at the 1,4-BQMCPE. By increasing the pH the reduction peak potential of TDZ shifts to more negative potentials up to pH 7.0 which is an indication of proton participation in the reduction of TDZ at acidic medium. But from pH 7.0 and above the peak potential remained constant which indicated the absence of protons during the reduction of TDZ at 1,4-BQMCPE in neutral and basic media.

The results revealed that voltammetric responses were strongly pH dependent in the acidic region in contrast to the neutral and alkaline mediums which was in agreement with most of the electrochemical methods reported [7, 9, 10]. A peak potential independent of pH in the neutral and alkaline medium could be attributed to the unavailability of protons that promote the reduction of TDZ at pHs larger than its pka value.

The reduction peak current increased sharply with an increasing pH value from 2 to 5 and then decreased when further increasing solution pH. Considering the determination sensitivity, pH 5 was chosen for the subsequent analytical experiments.

**Scheme 2:**
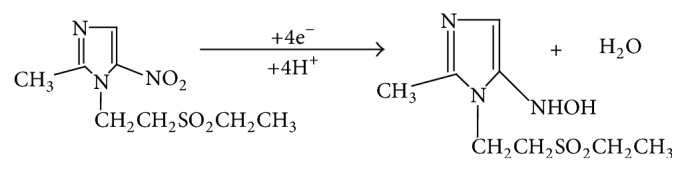
The proposed reduction reaction mechanism of tinidazole on 1,4-BQMCPE.

**Figure 5:**
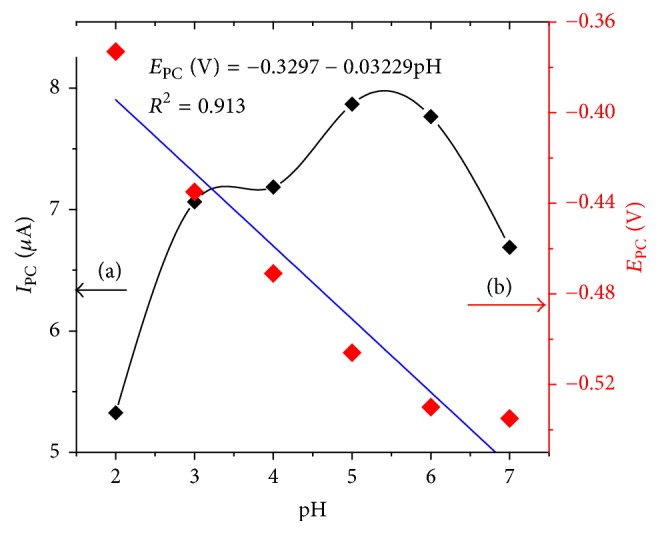
Plot of reductive (a) peak current and (b) peak potential versus pH for 0.5 mM TDZ in 0.1 M BRB SOLUTION of different pH values at 1,4-BQMCPE. Scan rate: 100 mV/s.

Since there was an indication of participating of protons during reduction of TDZ in the acidic medium, a linear relationship between the peak potential and solution pH (up to pH 7) (Figure 5(b)) with a linear regression equation and correlation coefficient of *E*_P_ (V) = −0.3297 − 0.03229pH and *R*^2^ = 0.913, respectively, was obtained. A slope of 0.03229 V/pH typically suggested that the number of protons taking part in the electrode reaction is equal to the number of electrons that participated in the rate determining step. Since the number of electrons that participated are already calculated in the previous discussion to be four, it is now possible to suggest that the irreversible reduction of TDZ at 1,4-BQMCPE involves four electrons and four protons (Scheme 2).

### 3.2. Differential Pulse Voltammetric Investigation

**Figure 6:**
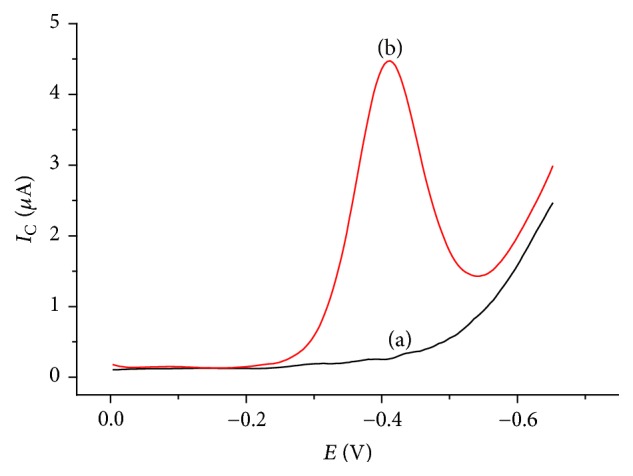
Differential pulse voltammograms of 1,4-BQMCPE in pH 5.0 BRB solution containing (a) no TDZ and (b) 0.5 mM TDZ.

The electrochemical reduction of TDZ at 1,4-BQMCPE was studied using differential pulse voltammetry. No peak was observed at the voltammogram of 1,4-BQMCPE in buffer solution while there was an irreversible reductive peak in the presence of TDZ (Figure 6).

Since the kinetics of the reduction of TDZ at 1,4-BQMCPE are adsorption controlled, the accumulation (*E*_acc_) and accumulation time (*t*_acc_) were optimized. Moreover, method parameters such as scan rate, pulse amplitude, and modifier composition were optimized.

#### 3.2.1. Optimization of DPV Parameters for TDZ Determination

**Figure 7:**
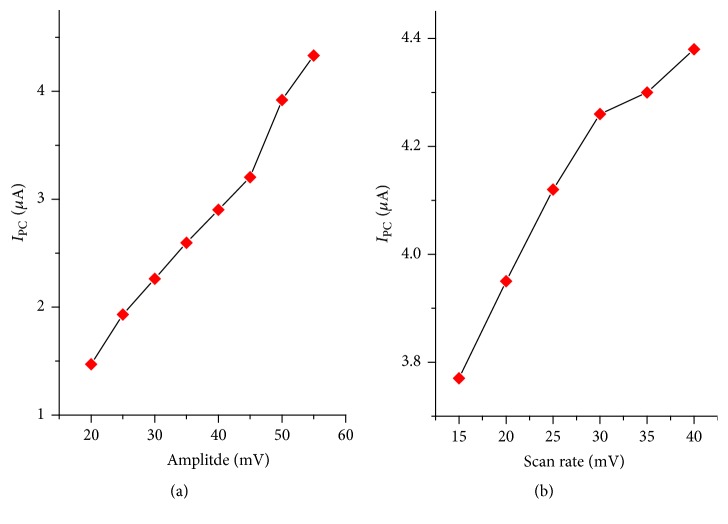
Plot of differential pulse voltammetric peak current response of 1,4-BQMCPE for 0.5 mM tinidazole (a) versus differential pulse amplitude and (b) versus scan rate at 45 mV of amplitude.

*DPV Pulse Amplitude and Scan Rate*. The effect of differential pulse amplitude and scan rate on the peak current of 0.5 mM TDZ in pH 5 BRB solutions was studied in the range of 20 to 55 mV and 15–40 mV/s, respectively. As shown in Figure 7, upon increasing both amplitude and scan rate, a linear increase in the peak current was observed accompanied by peak broadening in particular when the amplitude was greater than 45 mV. Thus, 45 mV of amplitude and 30 mV/s of scan rate were chosen as optimum values.

*Accumulation Potential (E*_*acc*_ *) and Accumulation Time (t*_*acc*_). The effect of accumulation potential (*E*_acc_) on the peak current for 0.5 mM TDZ was examined in the range of −50 to −400 mV. The peak current for TDZ initially increased when the accumulation potential was increased and reaches maximum at −300 mV as shown in Figure 9. Therefore, the accumulation potential (*E*_acc_) of −300 mV was chosen as the optimum accumulation potential for further work.

**Figure 8:**
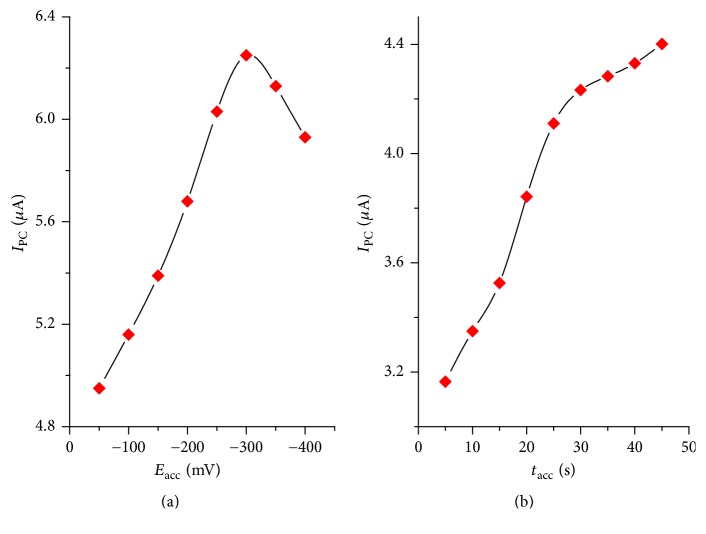
Plot of differential pulse voltammetric peak current response of 1,4-BQMCPE for 0.5 mM TDZ versus (a) accumulation potential and (b) accumulation time at *E*_acc_ = −300 mV.

The effect of accumulation time (*t*_acc_) on the peak current of 0.5 mM TDZ at a potential of −300 mV was also investigated. The variation of accumulation time between 5 and 45 s at an accumulation potential of −300 mV showed that the peak current increased with the increase in accumulation time up to 30 s and then almost leveled off (Figure 8(b)). The increase of peak current with increase in accumulation time indicated that TDZ can be accumulated at the surface of the 1,4-BQMCPE. The leveling off peak current after 30 s could be ascribed to the saturation of the surface of the electrode. So the accumulation time of 30 s was selected as an optimum accumulation time for further experiments.

**Figure 9:**
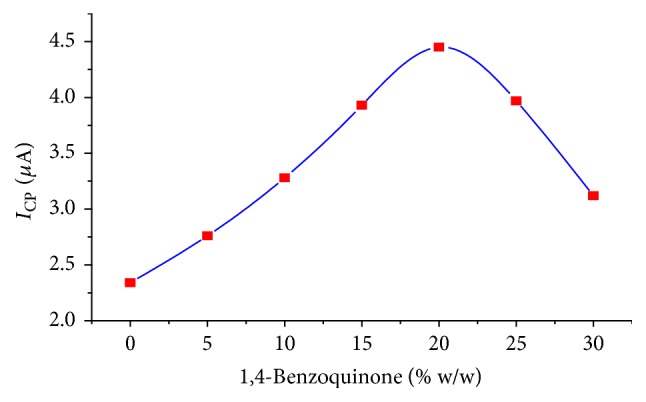
The effect of amount of benzo quinone (modifier) on the reduction peak current 0.5 mM tinidazole. Amplitude = 45 mV. Scan rate = 30 mV/s.

*Effect of Modifier Composition*. When the content of benzoquinone was increased from 0% to 15% (w/w), as can be seen from Figure 9, the peak current increased with increasing the modifier composition from 0% to to 20% (w/w). A peak current decrease was observed at modifier composition higher than 20% (w/w) and, hence, a modifier composition of 20% (w/w) was taken as the optimum modifier composition throughout the present work.

*Optimum Experimental Conditions*. The effects of experimental parameters have been studied to obtain optimum experimental conditions for differential pulse Voltammetric determination of TDZ at 1,4-BQMCPE. The optimum parameters used for the experiment are summarized in Table 1.

#### 3.2.2. Calibration Plot for TDZ

**Figure 10:**
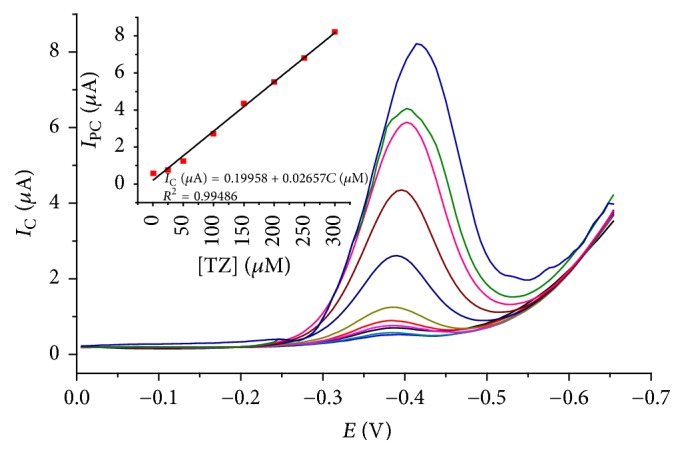
DPV of 1,4-BQMCPE (corrected for background) in pH 7 BRB solution of different concentrations of TDZ (1, 5, 25, 50, 100, 150, 200, 250, 350, 400, 450, and 500 *μ*M, resp.). Inset: calibration curve of peak current (*I*_PC_) versus concentration (*C*) of TDZ in BRB solution on 1,4-BQMCPE.

Under the optimized experiment conditions, the calibration curve for TDZ in pH 5.0 BRB at 1,4-BQMCPE was characterized by DPV. As can be seen (Figure 10 inset), the reduction peak current (*I*_PC_) was proportional to the concentration of TDZ in the range from 1 to 300 *μ*M. The calibration curve for ten average data points (*n* = 10) was found to be linear with *R* = 0.99486 and a regression equation of *I*_PC_ (*μ*A) = 0.19958 + 0.02657*C* (*μ*M). The limit of detection and limit of quantification were calculated to be 1.10 × 10^−7^ and 3.77 × 10^−7^, respectively.

The detection performance of 1,4-BQMCPE was compared with other electrodes and the results are listed in Table 2. As can be seen from the table, the developed method, which used a very cheap and easily available electrode, showed a relatively comparable limit of detection and linear range even with the methods used which are definitely much more expensive electrodes like SWCNT/GCE.

#### 3.2.3. Reproducibility and Stability of 1,4-BQMCPE

In this experiment, reproducibility was investigated by considering three modified electrodes prepared independently by taking triplicate measurements using the three electrodes. The reproducibility expressed in relative standard deviation was found to be 3.44% for 0.5 *μ*M TDZ solution showing excellent reproducibility of the method.

The 1,4-BQMCPE showed high stability. As it is shown during the experiment, there has been no significant difference in the peak current responses for the same electrode and the same standard solutions over a period of one month.

#### 3.2.4. Determination of TDZ in Pharmaceutical Tablets

The applicability of 1,4-BQMCPE for the determination of TDZ was demonstrated by applying it to determine the TDZ content in some pharmaceutical tablets. These samples were prepared as described in Section 2.2.2. Briefly, tablets from each brand (APF and EPHARM) were weighed and powdered. An amount corresponding to a stock solution of 0.01 M concentration was weighed and transferred into a 100 mL flask and completed to the volume with pH 5 BRB solution. Finally, 50 and 90 *μ*M tablet sample solutions for each brand were prepared from the corresponding stock solution. Differential pulse voltammograms were recorded following the outlined voltammetric procedure and optimized conditions as described earlier. Analyses were done using triplicate measurements and the mean values were recorded. The determination of TDZ in these samples was carried out according to the linear regression equation formulated for the calibration curve.

Table 3 presents the summary of the analyses on the TDZ composition of the two tablet brands. The tablet formulations for the collected two brands being 500 mg of TDZ per tablet, the amount of TDZ found using the developed method ranged between 479.845 and 491.95 mg per tablet showing deviation of the experimental result from the theoretical value. Lower levels of TDZ in tablets than prescribed value may be due to the possible mass loss of TDZ during preparation or some TDZ degradation during storage or this can be due to originally lower levels of TDZ in the tablets.

#### 3.2.5. Recovery Analyses

The applicability of the developed method for the determination of TDZ in real samples was further evaluated by recovery analyses of spiked standard TDZ in the two brand tablet samples. The differential pulse voltammograms for 90 *μ*M tablet samples of each tablet brand, each spiked with the same amount of standard TDZ (5 mg), were recorded. The % recoveries were calculated taking into consideration the amounts previously detected in the 90 *μ*M tablet samples. The recovery results are summarized in Table 4. The results confirmed the potential applicability of the developed method for TDZ analyses in real samples. As can be seen from Table 4, the maximum relative standard deviation (RSD) recorded for a duplicate measurement was 3.11 showing the reproducibility of the response of the electrode. The results obtained using the proposed method are almost comparable to or even better than the results obtained by a method which used a very expensive single-wall carbon nanotubes coated glassy carbon electrode which has a maximum RSD of 4.6 [7].

#### 3.2.6. Determination of TDZ in Human Urine

Approximately 20–25% of the administered dose of TDZ is excreted in the urine mainly as unchanged drug [29]. Therefore considering this the applicability of 1,4-BQMCPE for the determination of TDZ was also investigated by the determination of TDZ in human urine solution.

Taking into consideration the amount previously detected, sample solutions with concentration of 90 *μ*M in urine samples were prepared using the diluted human urine (as described in Section 2.2.3) for both TDZ tablet brands. Under the optimized parameters, DPV was employed to determine the reductive current response for the urine samples at 1,4-BQMCPE and the results summarized in Table 5 were obtained.

#### 3.2.7. Interference Analyses

Different potential interferents were taken to study selectivity of the method for TDZ. For the interference studies, drugs which could be present in the TDZ tablet (omeprazole and ciprofloxacin), drug which have structural similarities with TDZ (metronidazole), and ingredients (excipients) of TDZ drug were selected.

As can be seen from Table 6, the presence of metronidazole showed positive interference on the peak current for TDZ and the peak current change was greater than 5%, a comparable result with other reports [7, 30]. This is because they contain the same reductive groups that can be reduced near the potentials of TDZ [7]. But the presence of the other selected interferents did not significantly affect the peak current response for the TDZ and the change in peak current was less than 5%.

## 4. Conclusion

1,4-Benzoquinone modified carbon paste electrode was successfully applied for the voltammetric determination of TDZ in real pharmaceutical tablets and human urine. The proposed method provides a sensitive and simple approach for determination of TDZ in real pharmaceutical tablets and human urine. Because of its very low limits of detection and quantification, relative to the previously reported works which have used expensive electrodes, the proposed method could be applied in clinical laboratories and pharmacokinetic studies even in a complex matrix system like pharmaceutical formulations.

## Figures and Tables

**Table 1:** Optimum values of the experimental parameters for the determination of TDZ by differential pulse voltammetric (DPV) technique at 1,4-BQMCPE.

Parameter	Optimized value
pH of the buffer solution	5
DPV pulse amplitude (mV)	45
DPV Scan rate (mV/s)	30
Accumulation potential (mV)	−300
Accumulation time (s)	30
Effect of modifier composition (% (w/w))	20

**Table 2:** Comparison between the developed method and other reported methods.

Electrode	Method	Linear range (M)	Detection limit (M)	Ref.
SWNT/GCE	DPV	5 × 10^−8^–4 × 10^−5^	1 × 10^−8^	[7]
HMDE	DPP	2 × 10^−6^–1.1×10^−3^	9.7 × 10^−7^	[8]
GCE	DPV	10^−7^–10^−9^	3 × 10^−10^	[9]
CPE	DPV	5 × 10^−6^–2 × 10^−4^	5.06 × 10^−7^	[10]
1,4-BQMCPE	DPV	10^−6^–5 × 10^−4^	1.1 × 10^−7^	*This work*

**Table 3:** Amount of TDZ detected in two brands of tablets using the developed method.

Tablet	Solution	Expected (*μ*M)	Detected^*∗*^		Labeled value (mg/tablet)	Measured (%)
			In *μ*M	mg/tablet		
APF	a	50	48.174	481.74	500	96.35
	b	90	86.372	479.845	500	95.97
EPHARM	a	50	49.195	491.95	500	98.39
	b	90	87.538	486.32	500	97.26

^*∗*^Mean of triplicate measurements.

**Table 4:** Percentage recovery of TDZ from pharmaceutical tablets.

Tablet	Present TDZ (mg)	Added TDZ (mg)	Expected TDZ (mg)	Found (mg)^*∗*^	Recovery (%) ± % RSD
APF	0.534	5	5.534	5.396 ± 0.0023	97.51 ± 3.11
EPHARM	0.541	5	5.541	5.399 ± 0.0027	97.44 ± 2.71

^*∗*^Mean of double measurements.

**Table 5:** Percentage recovery of TDZ from human urine sample solutions.

Tablet	Present TDZ (mg)	Added TDZ (mg)	Expected TDZ (mg)	Detected (mg)	Recovery (%) ± % RSD
APF	0.556	5	5.556	5.299	95.37 ± 2.08
EPHARM	0.556	5	5.556	5.384	96.91 ± 3.29

**Table 6:** Interference study of TDZ with different selected drugs.

	Concentration	Recorded	Signal
	(in *μ*M)	Signal (*I*_P_/*μ*A)	Change (%)
*TDZ*	*200*	*5.512*	
Interferents added			
Cellulose	80	5.501	0.199
Magnesium striate	80	5.394	2.141
Titanium dioxide	80	5.407	1.905
Polyethylene glycol	80	5.351	2.921
Metronidazole	80	6.084	10.377
Omeprazole	80	5.473	0.708
Ciprofloxacin	80	5.297	3.900
